# Neurosurgical management of adult diffuse low grade gliomas in Canada: a multi-center survey

**DOI:** 10.1007/s11060-015-1949-0

**Published:** 2015-10-10

**Authors:** Osaama H. Khan, Warren Mason, Paul N. Kongkham, Mark Bernstein, Gelareh Zadeh

**Affiliations:** Division of Neurosurgery, University Health Network, Toronto Western Hospital, University of Toronto, 399 Bathurst Street, Toronto, ON M5T 2S8 Canada; Princess Margaret Hospital, 610 University Avenue Suite 18-717, Toronto, ON M5G 2M9 Canada

**Keywords:** Awake craniotomy, IDH-1, 1p19q, Wait-and-see, Practice patterns, Watchful waiting, Astrocytoma, Oligodendroglioma, LGG

## Abstract

Adult diffuse low-grade gliomas are slow growing, World Health Organization grade II lesions with insidious onset and ultimate anaplastic transformation. The timing of surgery remains controversial with polarized practices continuing to govern patient management. As a result, the management of these patients is variable. The goal of this questionnaire was to evaluate practice patterns in Canada. An online invitation for a questionnaire including diagnostic, preoperative, perioperative, and postoperative parameters and three cases with magnetic resonance imaging data with questions to various treatment options in these patients was sent to practicing neurosurgeons and trainees. Survey was sent to 356 email addresses with 87 (24.7 %) responses collected. The range of years of practice was less than 10 years 36 % (n = 23), 11–20 years 28 % (n = 18), over 21 years 37 % (n = 24). Twenty-two neurosurgery students of various years of training completed the survey. 94 % (n = 47) of surgeons and trainees (n = 20) believe that we do not know the “right treatment”. 90 % of surgeons do not obtain formal preoperative neurocognitive assessments. 21 % (n = 13) of surgeons and 23 % of trainees (n = 5) perform a biopsy upon first presentation. A gross total resection was believed to increase progression free survival (surgeons: 75 %, n = 46; trainees: 95 %, n = 21) and to increase overall survival (surgeons: 64 %, n = 39, trainees: 68 %, n = 15). Intraoperative MRI was only used by 8 % of surgeons. Awake craniotomy was the procedure of choice for eloquent tumors by 80 % (n = 48) of surgeons and 100 % of trainees. Of those surgeons who perform awake craniotomy 93 % perform cortical stimulation and 38 % performed subcortical stimulation. Using the aid of three hypothetical cases with progressive complexities in tumor eloquence there was a trend for younger surgeons to operate earlier, and use awake craniotomy to obtain greater extent of resection with the aid of cortical stimulation when compared to senior surgeons who still more often preferred a “wait-and-see” approach. Despite the limitations of an online survey study, it has offered insights into the variability in surgeon practice patterns in Canada and the need for a consensus on the workup and surgical management of this disease.

## Introduction

The management of adult diffuse low-grade gliomas (LGG) is variable. Mounting non-Class I evidence suggests that early upfront extensive microsurgical resection of LGGs is associated with a more favorable prognosis [1–18]. The level of evidence to support clinical care remains controversial and we are faced with the ongoing challenge of designing the best management strategy for individual patients. Controversies include: the necessary components of the diagnostic workup; the role of a “wait-and-see” strategy of following patients based on their clinical status and imaging alone; to the nature and goals of surgical intervention; and postoperative management issues including but not limited to imaging, role of repeat surgery, adjuvant treatment, and follow-up. The aim of this study is to gain insight into the practice patterns of neurosurgeons and trainees in Canada, where the vast majority of neurosurgery is centralized and performed at academic centers. We postulated that when compared to senior neurosurgeons, trainees and younger neurosurgeons have practice patterns that (i) utilize more tools in the workup and surgical management of patients (ii) believe that early surgery can impact outcomes (i.e. overall survival, progression free survival—PFS etc.) (iii) more readily incorporate awake craniotomy and mapping for surgical resections.

## Methods

A questionnaire was created in English and French on an online password protected survey system (www.surveymonkey.com). An email with a brief rationale for the study was sent to practicing neurosurgeons, neurosurgery fellows and residents across Canada registered at academic centers or working at hospitals with neurosurgical services. Over 90 % of neurosurgery in Canada is practiced at academic centers.

**Fig. 1:**
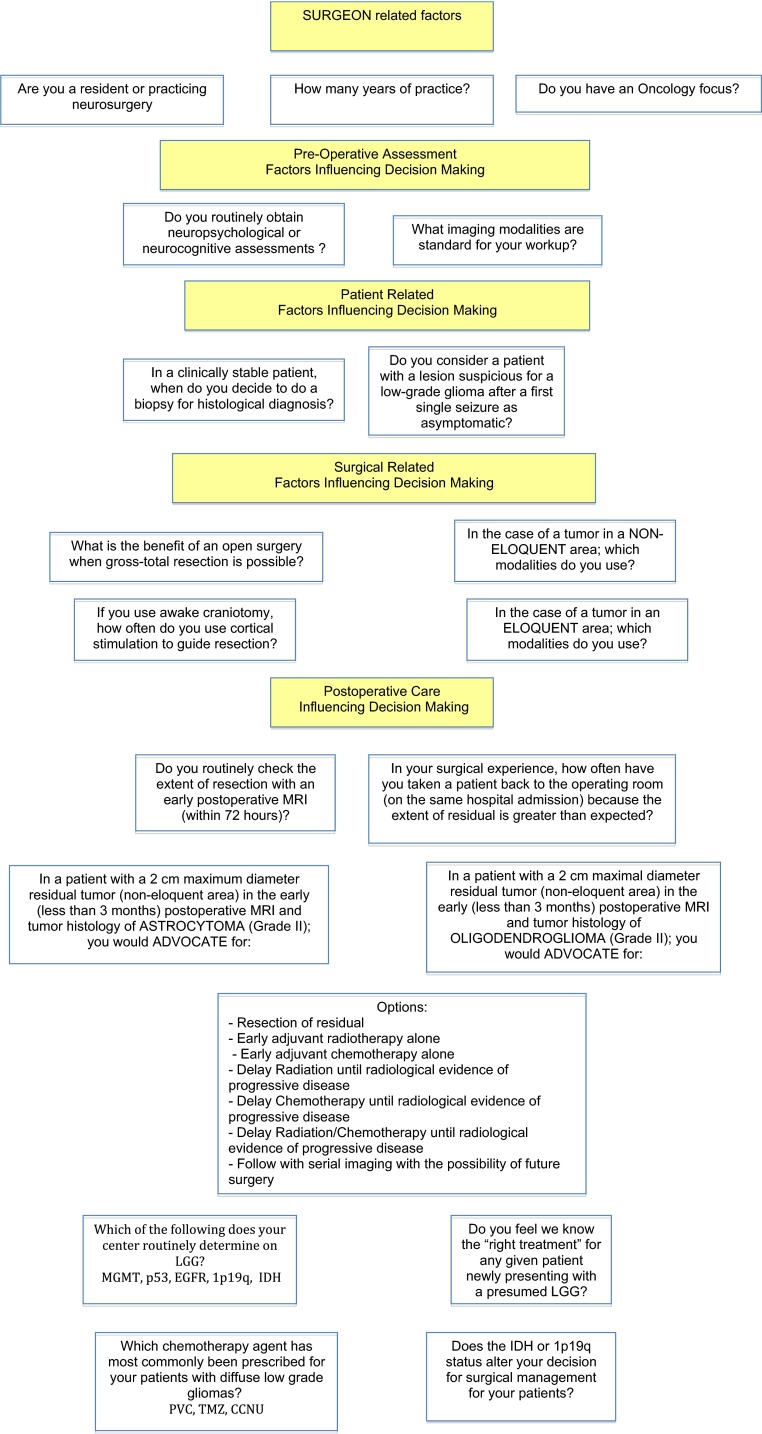
Flow diagram illustrating questions presented to practicing neurosurgeons and trainees in Canada. Survey conducted online using an online anonymous password protected questionnaire (SurveyMonkey; www.surveymonkey.com)

The questionnaire asked for symptomatology, preoperative diagnostic tools, wait-and-see strategy versus surgical treatment, intraoperative applications, and postoperative management. This was followed by three separate cases of patients with MRI images demonstrating low-grade lesions (non-enhancing), and with the question “How would you treat this patient?”. Figure 1 illustrates the flow diagram of questions. A diffuse LGG was defined in the survey as a World Health Organization (WHO) grade II entity, with surgically curative WHO grade I pilocytic astrocytomas excluded. Pure astrocytic and pure oligodendroglial tumors were used as examples to determine how histological subtyping possibly influences treatment decisions.

**Fig. 2:**
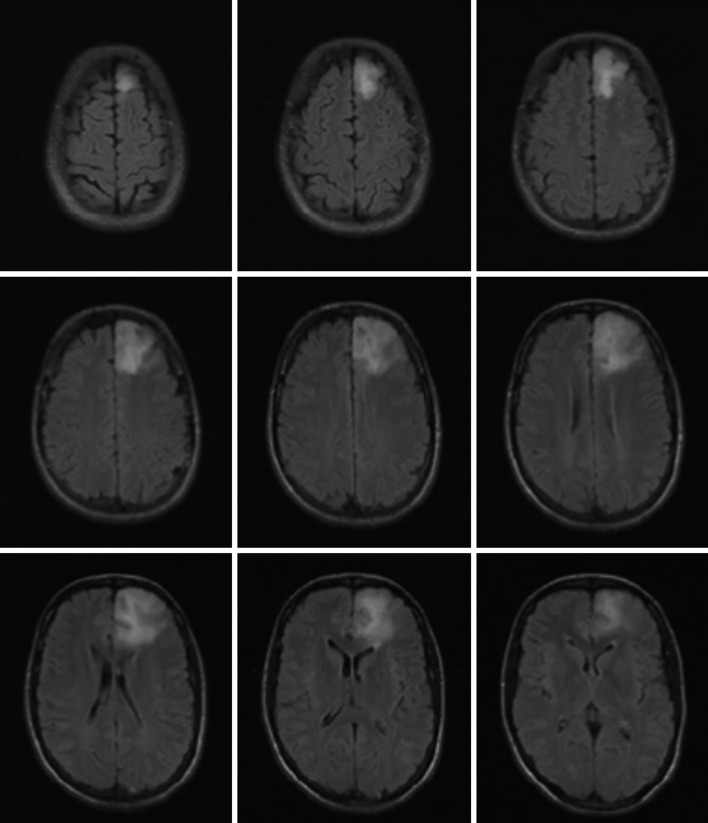
How would you treat this patient? A 24 year old right hand dominant female patient with a history of 2 generalized seizures. MRI FLAIR sequence is shown. There was NO enhancement with gadolinium

**Fig. 3:**
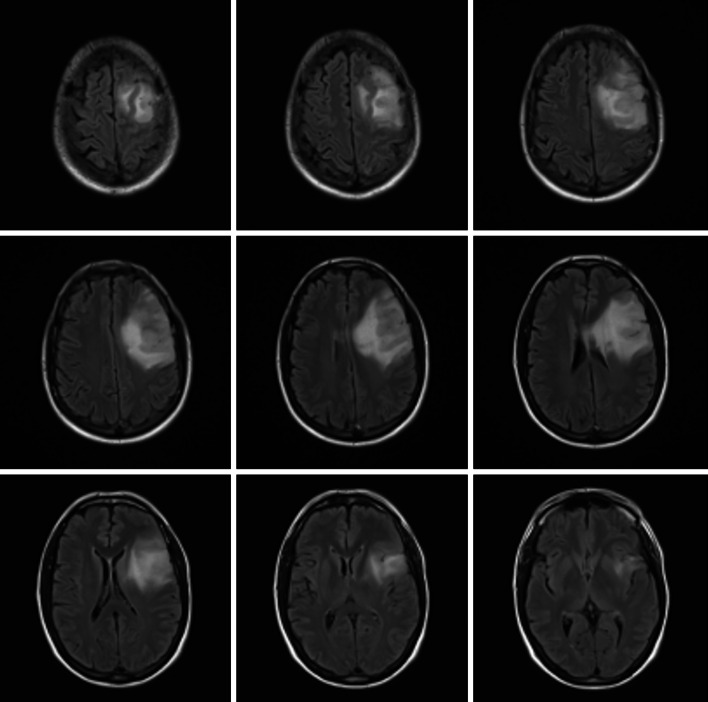
How would you treat this patient? A 52 year old male, right hand dominant, presents with simple partial seizures with motorized aphasia. Neurologically intact. MRI FLAIR sequence is shown. There was NO enhancement with gadolinium

**Fig. 4:**
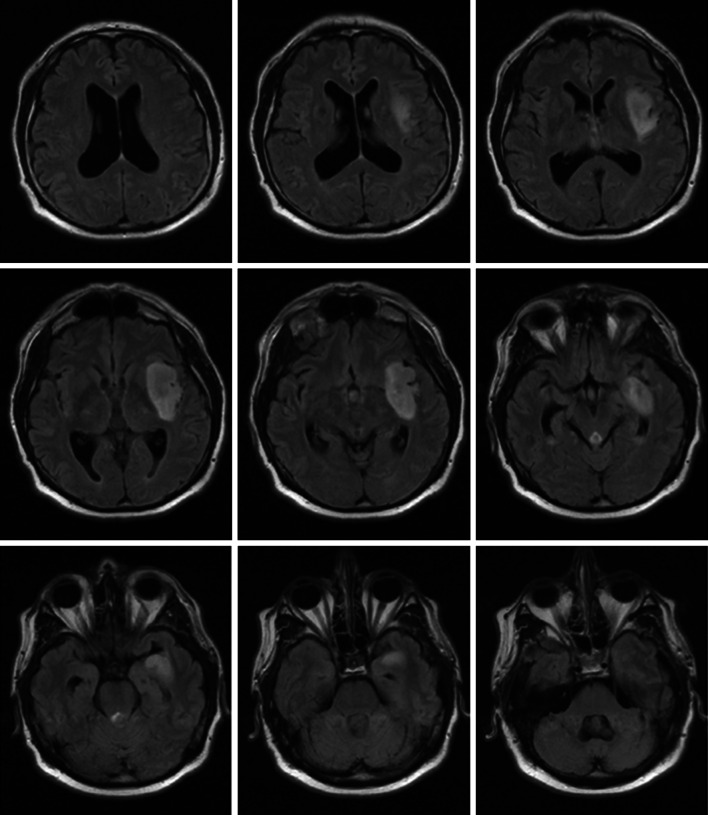
How would you treat this patient? A 49 year old male right hand dominant presents with complex-focal seizures, without any neurological deficit. MRI FLAIR sequence is shown. There was no enhancement with gadolinium

Authors developed the concept and design of the questionnaire. However, certain questions were adapted and/or incorporated, with approval from lead author and journal publisher, from a previously published survey completed in 2011 looking at strategies of high-volume German neurosurgical departments (not individual surgeons) [19]. Identical case examples from that survey and representative figures, were also used (Figs. 2, 3, 4).

## Results

A total of 356 emails were sent with 87 (24.7 %) responses collected over a 3 month period (March–May 2013). Sixty-five (75 %) responses were from practicing neurosurgeons while twenty-two (25 %) were from trainees. The majority of results discussed in the paper are therefore primarily based on responses from practicing surgeons unless otherwise stated. Relevant responses from surgeons and trainees are summarized in Table 1. Sixty-one responses (94 %) were completed in English and 4 (6 %) were completed in French. The range of years of practice was less than 5 years 22 % (n = 14), 5–10 years 14 % (n = 9), 11–15 years 14 % (n = 9), 16–20 years 14 % (n = 9), 21–25 years 14 % (n = 9), and 26 years or greater 23 % (n = 15). Thirty-one (48 %) stated that they considered their practice to have a neuro-oncology focus.

**Table 1:** Select responses of surgeons (n = 65) and trainees (n = 22) for management of LGGs

	Surgeon (%)	Trainee (%)
Consider first presentation of LGG with seizure as asymptomatic	26	41
Biopsy upon first presentation	21	23
Awake craniotomy alters surgical outcome	75	91
GTR increases progression free survival	75	95
GTR increases overall survival	64	68
Awake craniotomy for eloquent tumor	80	100
Cortical stimulation	93	83
Subcortical stimulation	38	14
Postop MRI <72 h	72	77
IDH or 1p19q alters surgical management	68	89
Do we know the “right treatment”?	94—no	94—no

Nighty-four percent of surgeons (n = 47) and trainees (n = 20) believe that we do not know the “right treatment” for any given patient presenting with a presumed newly diagnosed LGG.

### Clinical presentation and diagnostic workup

For a patient presenting solely with a *new onset* seizure with a lesion suspicious for a LGG, 26 % (n = 17) considered this individual to be asymptomatic while 74 % (n = 48) stated that this patient is symptomatic. When asked the same question but now with *recurrent* seizures, 12 % (n = 8) believed that this patient is asymptomatic while 88 % (n = 57) stated that this patient is symptomatic. MRI was considered a “standard” diagnostic workup for a LGG by 100 % (n = 62) of respondents, while MR Spectroscopy and Positron Emission Tomography (PET) were considered standard workup in 23 % (n = 14) and 3 % (n = 2), respectively. The vast majority of surgeons (90 %, n = 56) do not obtain neurocognitive assessments.

### Surgical management

Surgical options for the LGG patient include biopsy (frameless or frame-based needle biopsy, open surgical biopsy), and surgical resection. Biopsy alone was deemed appropriate in various circumstances. When given the opportunity to select *more then one response* for when the surgeon uses a biopsy in the context of LGGs; upfront biopsy on all first presentations for diagnostic purposes was selected by 21 % (n = 13). Biopsy for grossly unresectable tumor (i.e.: eloquent area) was suggested by 39 % (n = 24). Seventy-five percent (n = 46) of surgeons use a biopsy when there is demonstrated tumor growth, and for diagnosis in cases with tumor enhancement on MRI in 79 % (n = 48).

When gross-total resection is possible, responders chose from the following perceived benefits, with more than one benefit being allowed in the response : providing a histological diagnosis (surgeon: 93 %, n = 57; trainee: 91 %, n = 20), for seizure reduction (surgeon: 74 %, n = 45; trainee: 41 % n = 9), to increase PFS (surgeon: 75 %, n = 46, trainee: 95 %, n = 21), to increase overall survival (surgeon 64 %, n = 39, trainee: 68 %, n = 15), to decrease intracranial pressure (surgeon: 72 %, n = 44; trainee: 55 %, n = 12) and for cytoreduction before adjuvant therapy (surgeon: 77 %, n = 47; trainee: 77 %, n = 17). Thirty-six percent of responders (n = 22) did not believe that gross-total resection would benefit overall survival.

Responders were asked about the role of intraoperative surgical adjuncts in a *non-eloquent* tumor, again with more than one choice allowed. Navigation was used by all; intraoperative ultrasound was used by 32 % (n = 19); awake craniotomy by 13 % (n = 8) and intraoperative MRI by 10 % (n = 6). For *eloquently* located tumors, 97 % (n = 58) used navigation, 80 % used awake craniotomy (n = 48), 35 % (n = 21) used neurophysiological monitoring, 30 % used functional MRI (n = 18), 27 % (n = 16) used diffusion tensor imaging (tractography), and 8 % (n = 5) used intraoperative MRI.

Fifteen surgeons (25 %) did not perform awake craniotomy in their practice. The remainder that did stated that cortical stimulation was used always 38 % (n = 23), regularly 32 % (n = 19), seldom 5 % (n = 3) or never 0 % (n = 0). Subcortical stimulation was used always 7 %, (n = 4), regularly 18 % (n = 11), seldom 33 % (n = 20) or never 17 % (n = 10).

### Postoperative management

MRI within the 72 h postoperative time window was used by 72 % (n = 44) of surgeons. The remaining surgeons obtained imaging within 1 month (n = 2), 3 months (n = 14), and 6 months (n = 2).

#### Residual disease

Thirty-four surgeons (57 %) have never required to repeat surgery during the same hospital admission due to residual tumor greater than expected, while 37 % (n = 22) reported having required to repeat surgery during the same admission in less than five patients in their career. In a patient with a 2 cm maximum diameter residual tumor (*non-eloquent* area) in the early (less than 3 months) postoperative MRI and tumor histology of *astrocytoma*, 69 % (n = 35) would delay treatment, 27 % (n = 14) would reoperate, and 4 % (n = 2) would recommend radiotherapy. When asked the same question with a pathology showing *oligodendroglioma*, 56 % (n = 28) would delay treatment, 26 % (n = 13) would reoperate, 16 % (n = 8) would recommend chemotherapy, and 2 % (n = 1) recommend radiotherapy.

### Recurrent disease

In an eloquently located LGG after biopsy showing an *astrocytoma* in a patient with intractable seizures managed with two anti-epileptic drugs, 32 % (n = 16) would advocate for radiation therapy alone, 6 % (n = 3) for chemotherapy alone, the remaining surgeons would either delay intervention chemotherapy/radiation until the time of tumor progression (38 %, n = 19), or suggest a wait-and-see approach when seizures controlled to intervene (24 %, n = 12). When asked the same question for biopsy results showing an *oligodendroglioma*, 50 % (n = 25) would advocate for chemotherapy only, 4 % (n = 2) for radiotherapy only, 22 % (n = 11) would delay radiation/chemotherapy until tumor progression while 24 % (n = 12) would wait-and-see when seizures controlled to intervene.

Temozolomide was the most common chemotherapy prescribed by 92 % of respondents (n = 46), followed by procarbazine, lomustine, vincristine (PCV) by 6 % (n = 3).

#### Multidisciplinary care

Fifty-percent (n = 25) of the surgeons surveyed play an active role in decision making for the use of adjuvant treatment (chemotherapy and/or radiation) in their patients with 78 % (n = 40) stating that they review all of their LGG in at a multi-disciplinary tumor board.

### Molecular genetic analysis

Sixty-eight percent (n = 34) of surgeons state that the isocitrate dehydrogenase (IDH) or 1p19q status did not alter their decision for surgical management. When this response was subdivided into years of practice, senior surgeons were more likely to rely on these molecular markers than trainees and younger surgeons (trainee 11 %, n = 2; <10 years: 26 % n = 5; 11–20 years: 27 % n = 4; >21 years: 44 % n = 7).

1p19q loss of heterozygosity (LOH) status was the most routinely determined molecular marker requested (96 %, n = 47), followed by p53 mutation (45 %, n = 22), MGMT methylation status (39 %, n = 19), IDH1 mutation (37 %, n = 18) and finally EGFR mutation (14 %, n = 7).

### Cases

Case 1 (Fig. 2) illustrates a LGG in a left superficial frontal location in a 24 year-old right hand dominant female with a history of two generalized seizures. Upfront interventions recommended: biopsy 16 % (n = 8), a “wait-and-see” strategy 23 % (n = 12), awake craniotomy 61 % (n = 31). When subdivided into years of practice (Table 2) awake craniotomy was more common in trainees 67 %, (n = 12) and younger surgeons with 79 % (n = 15) in less than 10 years of practice, 60 % (n = 9) in 11–20 years of practice, and 41 % (n = 7) in >21 years of practice. A biopsy was more commonly suggested in older surgeons (27 % n = 4, 11–20 years; 18 % n = 3, >21 years) when compared to younger surgeons (5 % n = 1, <10 years) and trainees (11 %, n = 2). A “wait-and-see” strategy was more common in senior surgeons (42 % n = 7, >21 years) than in younger surgeons (16 % n = 3, <10 years; 13 % n = 2, 11–20 years) and trainees (22 %, n = 4).

**Table 2:** Three cases (Fig. 2, 3, 4) of increasing complexities were presented with multiple options of management shows results of younger (n = 23; <10 years), middle (n = 18; 11–20 years) and senior surgeons (n = 24; >20 years of practice)

	Case 1	Case 2	Case 3
Awake surgery	Trainee 67 %	Trainee 39 %	Trainee 39 %
	Younger 79 %	Younger 42 %	Younger 16 %
	Middle 60 %	Middle 27 %	Middle 13 %
	Senior 41 %	Senior 18 %	Senior 0 %
Biopsy	Trainee 11 %	Trainee 44 %	Trainee 28 %
	Younger 5 %	Younger 42 %	Younger 74 %
	Middle 18 %	Middle 73 %	Middle 73 %
	Senior 27 %	Senior 29 %	Senior 29 %
“Wait-and-see”	Trainee 22 %	Trainee 17 %	Trainee 33 %
	Younger 16 %	Younger 16 %	Younger 26 %
	Middle 13 %	Middle 0 %	Middle 20 %
	Senior 42 %	Senior 53 %	Senior 65 %

Senior surgeons are more inclined to choose a “wait-and-see” approach and less likely to perform awake surgery

Case 2 (Fig. 3) depicts a 52 year-old right hand dominant male, presenting with a history of simple partial seizures with transient expressive aphasia that is neurologically intact with imaging of a left inferior frontal LGG (in/near eloquent cortex). Upfront interventions suggested: Biopsy 47 % (n = 24), awake craniotomy 30 % (n = 15), a “wait-and-see” strategy 23 % (n = 12). When subdivided into years of practice (Table 2) awake craniotomy was more common in trainees (39 %, n = 7) and younger surgeons 42 % (n = 8) with less than 10 years of practice, 27 % (n = 4) in 11–20 years of practice, and 18 % (n = 3) in >21 years of practice. A biopsy was more commonly suggested in the 11–20 years of practice surgeons (73 % n = 11, 11–20 years) followed by young surgeons (42 % n = 8, <10 years) and trainees (44 % n = 8) and finally senior surgeons at 29 % (n = 5). A “wait-and-see” strategy was more common in senior surgeons (53 % n = 9, >21 years) than in younger surgeons (16 % n = 3, <10 years; 0 % n = 0, 11–20 years) and trainees (17 % n = 3).

Case 3 (Fig. 4) describes a 49 year-old right hand dominant male with complex-focal seizures, without any neurological deficit, and an MRI demonstrating a left insular LGG. Upfront interventions suggested: Biopsy 53 % (n = 27), awake craniotomy 10 % % (n = 5), a “wait-and-see” strategy 37 % (n = 19). When subdivided into years of practice (Table 2) awake craniotomy was not a common choice among surgeons with 16 % (n = 3) in <10 years of practice, 13 % (n = 2) in 11–20 years and 0 % (n = 0) in >21 years, but was the most popular with trainees 39 % (n = 7). A biopsy was less likely chosen in senior surgeons at 29 % (n = 5) and trainees (28 %, n = 5) when compared to younger surgeons (<10 years of practice) 74 % (n = 14) and surgeons with 11–20 years of practice 73 % (n = 11). A “wait-and-see” strategy was more common in senior surgeons (65 % n = 11, >21 years) than in younger surgeons (26 % n = 5, <10 years; 20 % n = 3, 11–20 years) and trainees 33 % (n = 6).

In all three cases not one individual decided to radiate or give chemotherapy prior to any surgical intervention.

## Discussion

Diffuse LGG, a WHO grade II glioma, is characterized with anaplastic transformation over a 10 year period on average [20–22]. As stated by the European guidelines, maximal resection is currently the first therapeutic option in LGGs [23]. American guidelines also recommend maximal safe surgical resection, however, observation is appropriate for select patients [24]. The best management strategy has yet to be defined and remains a topic of great controversy, with traditional practices supporting a “wait-and-see” conservative approach while more recent data supports aggressive upfront resection to delay anaplastic progression and improved quality of life [25]. Of note is the recent recognition of molecular subtypes of LGG, including IDH mutation and 1p/19q co-deletion, which are of increasing clinical relevance. No doubt in the future such markers will be incorporated into practice guidelines as molecular information becomes integral to tumor classification.

We chose to investigate the practice patterns of neurosurgeons in Canada by surveying a diverse cohort of trainee and practicing neurosurgeons. We were able to collect an array of information that establishes the current national practice patterns, ranging from workup, as well as pre-, intra-, and post-operative management, and inquire about surgical management in three hypothetical cases ranging in complexity. This study demonstrates that there is no uniform approach to the management of LGGs in Canada. The information provided by this cohort establishes concepts concerning (i) the prevalent variability in management of LGGs nationally, suggesting the importance for introducing best practice guidelines for this disease (ii) areas of (re)education to establish a comprehensive and uniform management strategy of LGGs (iii) provide an opportunity for directing future studies nationally. Understanding the existing practice patterns across Canada will help to guide further initiatives not only nationally but internationally.

### Clinical management

Recent advances in neuroimaging have allowed for earlier diagnosis of gliomas, in patients with minor symptoms, single seizures or even those who are asymptomatic (incidental discovery) [26]. In this study 26 % of neurosurgeons surprisingly were unaware that a seizure can be symptomatic of a LGG. A lack of appreciation of this important fact could potentially delay intervention with negative clinical consequences. In addition, several reports support the effectiveness of surgery at improving seizure control after resection [27, 28].

### Neurocognitive assessment

It is well recognized that a standard neurological examination is not accurate enough to objectively assess patients with a LGG [29, 30]. While not yet a standard of care, neurocognitive assessments allow for a more in-depth evaluation of subtle deficits that may be present prior to treatment in LGG patients, and serve as a valuable baseline with which to compare subsequent clinical changes following treatment or in the setting of disease progression. This is of importance in part due to the longer life expectancy observed in this population and the impact on quality of life the disease and ongoing treatment may have, as compared with the high-grade glioma population. In our study, 90 % of surgeons reported that they do not routinely obtain any neuropsychological or neurocognitive assessments. Importantly, more than 90 % of LGG patients experience at least some neurocognitive deficit (for example, working memory disorders) prior to any treatment [29]. In the future, clinical studies, focusing on systematic neuropsychological examinations directed at defining valid, reproducible and efficient batteries of neurocognitive testing for LGG patients will be needed.

### Intraoperative adjuncts

Our study showed that 50 % of neurosurgeons “seldom or never” use intraoperative subcortical stimulation to guide tumor resection. The rate of permanent neurological deficits have been shown to be significantly reduced with awake mapping with less than 2 % in a recent series using intraoperative stimulation, in comparison with 15–20 % of severe worsening in series with no mapping [31–33]. One possibility for the uncommon use of intraoperative stimulation to guide tumor resection is the unfamiliarity with the approach as well as interpretation of the proper responses, especially for subcortical stimulation. Additionally, the familiarity of anesthesiologists with awake surgery could also play a role in a surgeon’s choice for surgical approach. Overall, this result highlights the need to introduce more dedicated fellowships, and educational courses for both practicing and in-training surgeons to acquire the skill set and experience needed to perform awake mapping, with the most realistically effective strategy being to promote subspecialty fellowship training in this area. Furthermore, dissemination of existing studies and establishing standards of care by the neuro-oncology community to define the value of such adjuncts is valuable. An alternative approach to consider focusing the management of LGGs, at centers with the necessary subspecialty services, also referred to as “centers of excellence”. This approach will only be successfully adopted if as a community neuro-oncology recognizes the value and need for specialized centers managing LGGs.

### Wait-and-see, surgery and extent of resection

A complete discussion of the “wait-and-see” approach versus surgery and extent of resection is beyond the scope of this study. In brief, most of the available retrospective literature suggests a survival benefit from aggressive surgical resection [17, 34–36], although there are data that reported no difference [37]. Maximal safe resection may also delay or prevent malignant progression [10, 38, 39] and recurrence [2].

A “wait-and-see” strategy continues to be commonplace in Canada especially amongst senior neurosurgeons as observed in the three cases in the survey. A subgroup analysis revealed that in all three proposed cases, surgeons with more than 20 years of practice were more likely to choose a “wait and see” approach (case 1, 42 %; case 2, 53 %; case 3, 65 %) compared to surgeons with less than 10 years of practice (case 1, 16 %; case 2, 16 %; case 3, 26 %). Whether this is a reflection of skepticism in advantages of surgical resection is unclear, however confirms a “wait-and-see” strategy has been the traditional teaching that is propagated in practice in Canada as we see from this study. There is no doubt that observation is desirable when tumors invade areas such-as primary motor or the paracentral lobule; however, the evidence in favor of observation is waning for areas of non-eloquence (e.g. right frontal lobe).

The data on clinical approaches towards surgical management of LGGs is in evolution, and though level 1 evidence is lacking with respect to the best surgical and medical approach, there is accumulating literature to support a role for early surgical intervention and attempt at near complete resection. However, given lack of definitive data taken together with the fact that LGGs are relatively uncommon and the concept of promoting dedicated centers for treatment of LGG is not yet adopted widely, it is to be expected that surgeons who have maintained a practice pattern across a number of years (such as senior surgeons in this survey), might chose to continue to take a more conservative approach with a’wait-and-see’ strategy unless definitive evidence is forthcoming to compel a change in management. Centralization of management of LGG in dedicated multidisciplinary settings with necessary subspecialty expertise is important in the path to make a culture and paradigm change a reality.

The argument for a more aggressive surgical approach has been shown by Berger and others who have found that both the preoperative as well as the postoperative volume to be of prognostic significance for the time to progression [2, 40]. A recent Norwegian study had shown the significant difference of survival in those centers with a preference for resection than those selecting a biopsy and watchful waiting [41] and with no significant difference in health related quality of life [42]. It is possible that the preference of “younger” surgeons to be more “aggressive” in achieving surgical debulking of a LGG is a sign of changing tide from “senior” surgeons that towards the goal of extensive resection to significantly delay, if not avoid, anaplastic transformation of the glioma.

The rationale for GTR when safely achievable [43] is that MRI underestimates the actual spatial extent of LGG invasion even when they appear well-delineated, which suggests that an extended resection of a margin beyond MRI-defined abnormalities, might improve outcome. This can be achieved in some cases by real time feedback by awake surgery with cortical and subcortical stimulation using such tasks as described earlier. However, recent studies looking specifically at oligodendroglioma show that extent of resection does improve PFS and overall survival but did not influence time to malignant transformation [44]. Clearly, more studies from single and multi-centers are required to further evaluate extent of resection in the various pathologies of LGGs.

A meta-analysis on the impact of brain mapping on glioma surgery outcome clearly showed that there were fewer severe neurological deficits and more extensive resections in tumors within eloquent regions when brain mapping was used [45]. In our view, the evidence is overwhelming that mapping, asleep or awake, during surgery for gliomas in eloquent regions should become the standard of care. The results of this study show that younger surgeons are more likely to perform awake surgery, underscoring the importance of including this surgical approach as a mainstay of residency and fellowship training. Furthermore, local seminars or courses provided by leaders in the field should be made available to practicing surgeons on the technical nuances of awake surgery, anesthesia, cortical and subcortical mapping, and intraoperative tasks.

### Adjuvant therapy

Temozolomide was the chemotherapy of choice in 92 % of respondents. Studies suggest that temozolomide has efficacy in the treatment of recurrent LGG, with response rates of 25–56 % [46, 47]. A recent study evaluated both quality of life and neurocognition of patients who were treated with a combination of chemotherapy and surgical resection for a LGG, showing an excellent tolerance of combined therapies [48]. Some have also proposed the use of preoperative chemotherapy for recurrent disease to allow for more extensive surgery [49].

Radiation therapy for LGG has been associated with impaired cognitive and executive function [50]. Two seminal randomized trials from the European Organization for Research and Treatment of Cancer (EORTC) have provided evidence to guide the postoperative management of low-grade glioma. Both trials, EORTC 22844 [51] and 22845 [52] showed no benefit of overall survival (OS), but the latter did show improved PFS and supported the option of delaying the use of postoperative therapy for a select group of patients. More recently updated results of RTOG 9802, a phase III trial that randomized higher risk patients (and observed lower risk patients) with LGG to fractionated radiotherapy plus or minus 6 cycles of postradiation PCV demonstrate a substantial improvement in overall survival in the PCV arm (13.3 vs 7.8 years) [53]. High-risk was defined as patients with diffuse gliomas (regardless of histology) who were 40 years or older with any extent of resection and patients who were 18 years or older whose tumors were less than completely resected. The subset of patients with LGG most likely to benefit from adjuvant postoperative radiotherapy has not been defined, and there is a lack of consensus about which patient- and tumor-specific factors confer a higher risk of progression. For our survey not a single surgeon decided to radiate or give chemotherapy prior to any surgical intervention in the three hypothetical cases.

When provided with a scenario of a patient with intractable seizures on two antiepileptic medications with an eloquent tumor of (i) *astrocytoma* histology, 62 % choose to delay any adjuvant therapy (includes delay radiation, chemotherapy and the “wait-and-see” cohort). However when the histology was (ii) *oligodendroglioma*, 50 % recommend upfront chemotherapy, 4 % for upfront radiation, while 46 % delayed any adjuvant therapy (includes delay radiation, chemotherapy and the “wait-and-see” cohort). There are several issues for discussion here. First we see the preference to observe a LGG rather than provide adjuvant therapy when surgery is not an option. Despite the low toxicity of chemotherapy (as described above) or the ability of radiotherapy to impede growth, the choice to avoid any intervention seems controversial. Second, the high preference of surgeons to select chemotherapy for oligodendroglioma but not astrocytoma histology was unexpected. This could be related to the respondents inferring the established sensitivity of anaplastic oligodendroglioma (WHO grade III) to chemotherapy [54, 55], however no current guidelines support the preference of treatment of based upon histology in LGGs [55].

### Recurrence

A large study, done in conjunction with the French Low-Grade Glioma Consortium, showed that anaplastic transformation can be significantly delayed after a more aggressive resection, and thus change the natural history of the disease. The authors also demonstrated that the resection of recurrent LGG significantly influenced survival in a multivariate analysis [17, 39].

The fact that LGG are slow-growing lesions allows the brain time to undergo plasticity in certain areas (i.e. language) [56–60]. The role of multistage surgical approaches makes it possible for a LGG removal in critical regions traditionally considered as unresectable, such as Broca’s area, Wernicke’s area, and the insular lobe (even in the “left dominant” hemisphere) [61–63]. This is explained by our better understanding of the plasticity phenomena and the functional reshaping (verified by intraoperative awake mapping) [59]. When asked about re-operation for recurrence (max diameter 2 cm), younger surgeons were more likely to follow with serial imaging (68 %) than compared to senior surgeons (31 %). Its unclear if this was based on the size of the recurrence, or whether younger surgeons were implementing this phenomenon of plasticity and intervening at a later time point though the concept of plasticity in adults remains yet to be established definitively.

### Limitations

This anonymous, online survey offered a wide spectrum of data from surgeons and trainees of varying areas of interest, expertise and practice patterns and provided data that allowed for discussion of timely relevant and controversial topics. However, there is no doubt that shortcomings exist for this study. The overall response rate for the survey was 24.7 %, the response from actively practicing neurosurgeons was 40 % (65 out of 160 actively practicing academic neurosurgeons). It should be noted that a lower response rate to this survey reflects the fact that we approached all neurosurgeons across all subspecialties. We note that those without a dedicated neuro-oncology practice were less inclined to respond to a specialty specific survey on the topic of LGGs. The response rate for neuro-oncology neurosurgeons is likely to be higher though a precise number can not be quoted given variability in definition of a subspecialty neuro-oncology focussed neurosurgeon. Besides sending repeat emails to non-responders further options need to be explored to improve survey response rates. Another shortcoming is that some of the techniques and technologies such as intraoperative MRI, awake craniotomy intraoperative mapping are not available at all centers and their unavailability might therefore have influenced some of the responses.

## Conclusion

In lieu of the results of planned and ongoing studies, it may be useful to outline specific recommendations for the management of LGG by either meta-analysis or an expert consensus interpretation of the available literature [64]. The reality is that a surgical treatment has an important role but will not in itself be curative for these diffusely infiltrating tumors. Despite mounting evidence that surgical intervention may influence the natural history of the disease in LGG patients, the ultimate “cure” will be in the realm of non-surgical modalities, and may eventually become tied to the specific molecular subtype of the tumor. Until that time we need to provide the best possible management for this devastating disease. Further focus groups on optimum treatment are needed [65]. Furthermore, our hope is that the survey results will provide a forum to engage clinicians involved and interested in treating LGG to form a nation-wide working-group that can focus on identifying further areas of investigations.
